# Neuromuscular regulation in zebrafish by a large AAA+ ATPase/ubiquitin ligase, mysterin/RNF213

**DOI:** 10.1038/srep16161

**Published:** 2015-11-04

**Authors:** Yuri Kotani, Daisuke Morito, Satoru Yamazaki, Kazutoyo Ogino, Koichi Kawakami, Seiji Takashima, Hiromi Hirata, Kazuhiro Nagata

**Affiliations:** 1 Laboratory of Molecular and Cellular Biology, Faculty of Life Sciences, Kyoto Sangyo University, Kyoto 606-8397, Japan; 2 Department of Cell Biology, National Cerebral and Cardiovascular Center, Suita 565-8565, Japan; 3 Department of Chemistry and Biological Science, School of Science and Engineering, Aoyama Gakuin University, Sagamihara 252-5258, Japan; 4 Center for Frontier Research, National Institute of Genetics, Mishima 411-8540, Japan; 5 Division of Molecular and Developmental Biology, National Institute of Genetics and Department of Genetics, Sokendai (The Graduate University for Advanced Studies), Mishima 411-8540, Japan; 6 Department of Medical Biochemistry, Graduate School of Medicine, Osaka University, Suita 565-0871, Japan; 7 CREST, Japan Science and Technology Agency, Saitama 332-0012, Japan

## Abstract

Mysterin (also known as RNF213) is a huge intracellular protein with two AAA+ ATPase modules and a RING finger ubiquitin ligase domain. Mysterin was originally isolated as a significant risk factor for the cryptogenic cerebrovascular disorder moyamoya disease, and was found to be involved in physiological angiogenesis in zebrafish. However, the function and the physiological significance of mysterin in other than blood vessels remain largely unknown, although mysterin is ubiquitously expressed in animal tissues. In this study, we performed antisense-mediated suppression of a mysterin orthologue in zebrafish larvae and revealed that mysterin-deficient larvae showed significant reduction in fast myofibrils and immature projection of primary motoneurons, leading to severe motor deficits. Fast muscle-specific restoration of mysterin expression cancelled these phenotypes, and interestingly both AAA+ ATPase and ubiquitin ligase activities of mysterin were indispensable for proper fast muscle formation, demonstrating an essential role of mysterin and its enzymatic activities in the neuromuscular regulation in zebrafish.

Mysterin (also known as RNF213) was originally isolated as a susceptibility gene for moyamoya disease, a human cryptogenic cerebrovascular disease characterized by progressive stenosis of the intracranial internal carotid artery and hemorrhage from characteristic collateral small vessels^1^. The mysterin gene is conserved among vertebrates and is ubiquitously expressed in human and mouse tissues^1^. This gene encodes a large (5,207 amino acids) cytoplasmic protein that contains two AAA+ ATPase modules and a RING finger ubiquitin ligase domain^1^,^2^. We and others have demonstrated that a rare single nucleotide polymorphism (SNP) causing an Arg to Lys missense mutation (R4810K) elevates the risk of moyamoya disease by more than 100-fold^1^,^3^. The mutation is involved neither in AAA+ ATPase modules (2397–2628 and 2738–2987) nor RING finger domain (3997–4093), while its pathological role has not yet been definitely identified.

AAA+ ATPase proteins typically form a hexameric toroidal complex that mediates various mechanical and physical intracellular events such as protein unfolding and membrane fusion following ATPase-dependent structural changes^4^. Indeed, mysterin forms a huge toroidal complex (~3.5 MDa), which is visible by electron microscopy (EM), and exhibits an ATPase activity *in vitro*^2^. The RING finger ubiquitin ligase covalently modifies substrate proteins with a small protein, ubiquitin, typically leading to proteasome-dependent protein degradation^5^. However, the substrate and biological relevance of the ubiquitin ligase activity of mysterin remain unknown. Intriguingly, mysterin is the only known protein that contains both AAA+ ATPase and ubiquitin ligase domains. Mysterin thus potentially coordinates two different enzymatic activities and may play a unique biological role.

We assumed that mysterin functions in the physiological structure and/or function of blood vessels because a missense SNP in mysterin drastically elevates the risk of moyamoya cerebrovascular disease. To examine this possibility *in vivo*, we previously performed antisense-mediated knockdown of a mysterin orthologue in zebrafish that express green fluorescent protein (GFP) in vascular endothelial cells, enabling the visualization of blood vessels in developing animals. Knockdown of a mysterin orthologue caused excess angiogenesis and misrouting of the vascular network in zebrafish, ensuring the link between pathological and physiological roles of mysterin in blood vessels^1^. However, the involvement of the AAA+ ATPase and/or ubiquitin ligase activities of mysterin in its function has not been elucidated, and the physiological significance of mysterin other than in blood vessels remains unexplored.

Here, we analyzed loss-of-function of mysterin from a broader perspective. Ubiquitous expression pattern of mysterin in animal tissues suggests its role in other than blood vessels. We found that suppression of a mysterin orthologue causes severe motor dysfunction in zebrafish embryos and larvae. Histological examinations revealed morphological anomalies in motoneurons and muscles. Our muscle-specific restoration of certain mysterin function further demonstrated that both AAA+ ATPase and ubiquitin ligase activities of mysterin are essential for muscle development *in vivo*.

## Results

### Reduced mysterin expression leads to impaired motility of zebrafish

**Figure 1:**
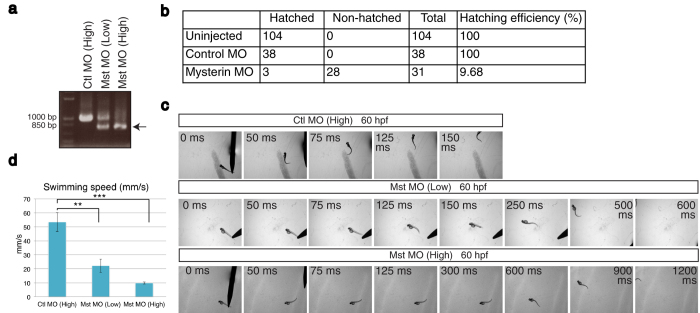
Suppression of mysterin-α leads to the failure of hatching and motor deficits in zebrafish. (**a**) Examination of the knockdown efficiency in morphants. Treatment with a low (1.7 ng) or high (5.1 ng) dose MO targeting mysterin-α (Mst) confirmed dose-dependent suppression of mysterin gene splicing at 2 dpf, as determined by RT-PCR. The lower band represents the products of impaired splicing (arrow). (**b**) Hatching defects in mysterin morphants. Morphants injected with a high dose of MO exhibit a significant hatching failure at 3 dpf. (**c**) Escape response to tactile stimulation at 60 hpf. Injection of a MO targeting mysterin reduces the movement of larvae in a dose-dependent manner. (**d**) Quantification of the swimming speed (mm/s) measured in 6 control morphants, 7 low-dose morphants, and 5 high-dose morphants at 60 hpf. **P < 0.01; ***P < 0.001.

Humans have a single mysterin gene, while zebrafish have two mysterin paralogues (α and β). Although these paralogues share a high sequence similarity, especially in their AAA+ ATPase and RING finger domains (Supplementary Fig. S1), mysterin-α is expressed to a greater extent than -β and is the primary isoform essential for angiogenesis in zebrafish cranial and trunk blood vessels. Indeed, knockdown of the β isoform did not cause any apparent vascular phenotypes^1^. To assess the *in vivo* function of the mysterin gene other than in angiogenesis, we injected antisense MOs into fertilized zebrafish eggs to block splicing of mysterin-α, thereby suppressing mysterin-α expression. Injection of 1.7 ng and 5.1 ng of the splicing-blocking MO dose-dependently suppressed mysterin mRNA splicing (1.7 ng: ~50%; and 5.1 ng: ~90%; Fig. 1a). Strong suppression of mysterin-α led to reduced hatching from chorions and an apparent developmental delay, which was confirmed by head-trunk angles. The angle between the first line drawn through the middle of the ear and the eye and the second line parallel to the trunk notochord (head-trunk angle) increases between 20 hr and 70 hr as a result of body straightening during embryogenesis^6^. The head-trunk angle of control embryos was 166.4° ± 1.2° (n = 14), whereas that of morphants was 137.1° ± 2.6° (n = 10), suggesting that there is a significant developmental delay in morphants at 3 day post-fertilization (dpf) (Supplementary Fig. S2a,b). Control embryos hatched at 2–3 dpf, whereas most morphants remained in chorions at 3 dpf (Fig. 1b). The morphants showed decreased motor activities *in ovo*, and this was thought to be the major cause of the reduced hatching efficiency. Therefore, we manually removed chorions at 1 dpf and assayed the touch-evoked motor response at 60 hr post-fertilization (hpf). Tactile stimulation triggered rapid swimming in control MO-injected larvae, while mysterin morphants exhibited a significant reduction in swimming velocity (control: 53.5 mm/s, n = 6; low-dose MO: 22.2 mm/s, n = 7; high-dose MO: 9.8 mm/s, n = 5; Fig. 1c,d; Supplementary Movie 1–3; Supplementary Fig. S2c). Thus, mysterin is necessary for motor function in zebrafish larvae.

### Axonal projection of primary motoneurons is impaired by mysterin suppression

**Figure 2:**
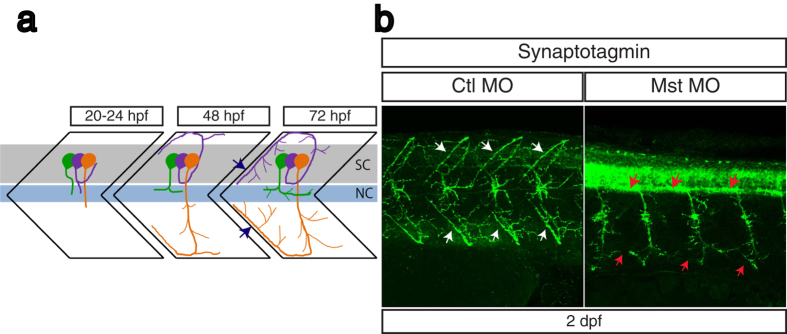
Suppression of mysterin-α leads to impaired projection of primary motoneurons during early development. (**a**) Schematic representation of motoneuron projection in somites during the early development of zebrafish. Primary CaP (orange), MiP (purple), and RoP (green) motoneurons project axons from the spinal code (sc). Each primary motoneuron projects its axon to a different area of muscle. In particular, intersegmental axonal projection of MiP and CaP motoneurons is indicated by blue arrows. The notochord is indicated by nc. (**b**) Motoneuron projection in the trunk region of a mysterin-α-suppressed animal (high dose). Primary motoneurons at 2 dpf are stained with an anti-synaptotagmin antibody (znp-1). The control animal exhibits proper projection of CaP and MiP motoneurons along the myotome segment and branched axons into intersegmental areas (white arrows). The morphant exhibits immature projection of CaP and MiP motoneurons along the myotome segment and reduced branched axons (red arrows).

Since motor function is mainly executed by motoneurons and muscle, we first examined the morphology of primary motoneurons in mysterin-α morphants. Three primary motoneurons exist in each embryonic somite, namely, rostral primary (RoP), middle primary (MiP), and caudal primary (CaP) motoneurons, which project to the transversal, dorsal, and ventral myotomes, respectively (Fig. 2a)^7^,^8^,^9^. Immunolabeling of these primary motoneurons with an anti-synaptotagmin antibody (znp-1) revealed the impaired projection of MiP and CaP motoneurons at intersegmental sites at 2 dpf in mysterin-α morphants treated with a high dose of MO, but not in control animals (Fig. 2b). In addition, the RoP projection into the myoseptum appeared to be immature in mysterin-α morphants. As described later, this compromised motor projection was cancelled when human mysterin was ectopically expressed by fast muscles (Fig. 4c and Supplementary Fig. S5). However, ectopic mysterin expression in fast muscle did not restore overall developmental delay in morphants, suggesting that the neuronal phenotype observed herein is attributable to specific impairment of neuromuscular regulation and not to general developmental retardation. Thus, mysterin-α plays an important role in primary motor projections, which accounts for the motor defects in mysterin-α-deficient zebrafish larvae.

### Myogenesis is impaired by mysterin knockdown

**Figure 3:**
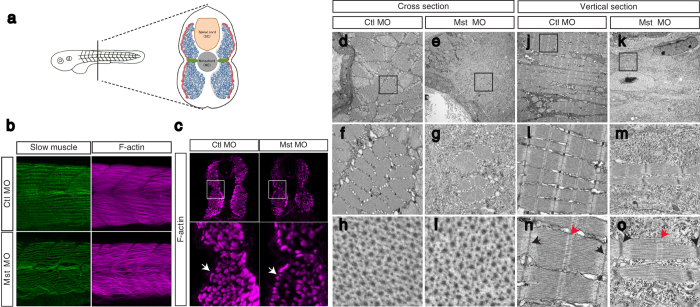
Suppression of mysterin-α leads to impaired muscle fiber formation in fast muscle. (**a**) Muscular composition of zebrafish embryos at 2 dpf. Slow muscle cells (red) form a monolayer structure beneath the skin. By contrast, fast muscle cells (blue) occupy the deeper region. MPCs (green) are located lateral to the notochord. (**b**) Fast and slow muscle structures. Fast and slow muscles in the trunk are stained with phalloidin, which recognizes actin fibers of both muscle types, and an anti-myosin heavy chain antibody (F59), which labels slow myosin fibers. F59 labeling revealed normal slow muscle fibers in control and mysterin morphants (left panels). Phalloidin labeling revealed an impaired fast muscle structure in morphants (right panels). (**c**) Cross-section of the trunk region. Slow and fast muscle structures are stained with phalloidin at 2 dpf. The density of F-actin fibers in the fast muscle region is severely decreased in morphants treated with a low dose of MO. White arrows indicate the slow muscle monolayer, which appears to be normal in morphants treated with a low dose of MO. (**d**–**o**) EM of the muscle structures of control animals and morphants treated with a low dose of MO at 2 dpf. Cross-sections (**d**–**i**) and vertical sections (**j**–**o**) of a control animal and morphant. The orientation of actin and myosin is shown in (**h–i**). The sarcomeric structure is shown in (**n–o**). The M-line and Z-disk are indicated by red and black arrows, respectively.

The other major component of motor function is skeletal muscle^10^. Skeletal muscle consists of slow and fast muscles, which are separately distributed in the zebrafish trunk (Fig. 3a)^11^. We observed slow and fast muscle structures using fluorescently labeled phalloidin, which labels actin fibers in both slow and fast muscles, and an anti-myosin antibody (F59), which recognizes myosin fibers in slow muscle. Although injection of a low dose of MO did not significantly affect the slow muscle structure (Fig. 3b, left panels), it severely altered the morphology of fast muscle such that it appeared abnormally loose and wavy (Fig. 3b, right panels). Closer observation of muscles in cross-sections confirmed that fast muscle was thin and loosely distributed in morphants, whereas superficial slow muscle was unaffected (Fig. 3c, white arrow). These results suggest that muscle fibers were significantly impaired in fast muscle but not in slow muscle, and that the thin muscle fibers lost their stiff structure and became fragile. To further observe the precise difference between control and morphant animals, we compared electron micrographs of their trunk regions. Cross-sections and vertical sections of fast muscle revealed thinner muscle fibers and enlarged interspace regions in the morphant trunk (Fig. 3d–m). This thin structure of muscle fibers was caused by a decreased number of myofibrils in morphants (control MO: 19.0 ± 1.4/5 μm^2^ square, n = 12; mysterin MO: 10.6 ± 1.6/5 μm^2^ square, n = 11, *P* < 0.001; Fig. 3d,e, and Supplementary Fig. S3a), suggesting that suppression of mysterin-α leads to impaired bundle formation in fast muscle. In a myofibril, the microstructures of actin (thin) and myosin (thick) filaments did not appear to be altered (Fig. 3h,i), while the sarcomeric Z-disc appeared to be thicker in morphants (Fig. 3n,o). In contrast to the abnormal structures of fast muscle, the slow muscle appeared normal in morphants (Supplementary Fig. S3b,c). These results suggest that the MO-induced motor deficit is attributable to morphological malformation of fast muscle fibers in addition to the impairment of motor projections.

### Recovery of fast muscle malformation by human mysterin

**Figure 4:**
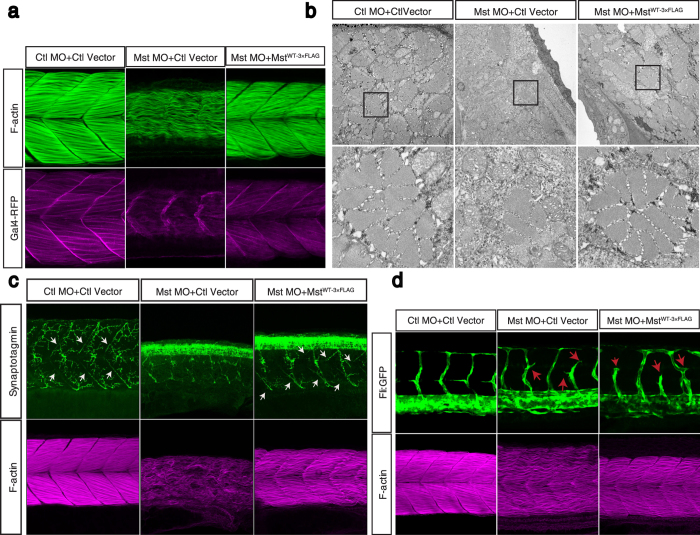
Fast muscle-specific expression of human mysterin restores the fast muscle malformation in morphants. (**a**) Ectopic expression of human mysterin restores the morphology of fast muscle in mysterin morphant larvae. Labeling of F-actin shows the formation of robust muscle fibers upon ectopic expression of mysterin in fast muscle, but not in the control. Expression of RFP driven by the UAS promoter shows that these larvae carry the GAL4FF-expressing transgene in fast muscle. (**b**) EM observation of restored fast muscle in the trunk. The lower panels show magnified images. (**c**) Projection of primary motoneurons in somites. Projection of motoneurons is impaired in mysterin-α morphants and restored by fast muscle-specific expression of human mysterin (white arrows). (**d**) Effect of suppression of mysterin-α and fast muscle-specific expression of human mysterin on vascular formation. Human mysterin tagged with a 3×FLAG epitope (WT-3×FLAG) is expressed in transgenic zebrafish that express GFP in vascular endothelial cells. Green (GFP) and magenta (F-actin) show trunk vessels and fast muscle, respectively.

The present results suggest that mysterin is essential for the proper development of fast muscle and motoneurons, in addition to that of blood vessels^1^. How does mysterin play an essential role in this tissue formation? To address this question, we attempted to rescue the developmental abnormalities by tissue-specific expression of human mysterin in mysterin morphants. Mysterin is conserved in vertebrates, and human and zebrafish mysterin share a high sequence similarity, especially in their AAA+ ATPase and RING finger domains (Supplementary Fig. S1). To express human mysterin in a tissue-specific manner, we generated a zebrafish transgenic line *Tg (gSA2AzGFF598A)* that expresses the modified transcriptional activator Gal4FF in the fast muscle fibers (see Methods). To express mysterin in fast muscle, we attempted to integrate a transgene, in which human mysterin containing a 3×FLAG epitope at its C-terminus is driven by the UAS promoter, into the zebrafish genome using the Tol2 transposon method (see Methods). Fast muscle-specific expression of Gal4FF was monitored by red fluorescent protein (RFP) expression in the fast muscle, while expression of mysterin-3FLAG was verified by immunoblotting with an anti-FLAG antibody (Supplementary Fig. S4a–c). As expected, fast muscle morphology in mysterin morphants was successfully restored when human mysterin was transiently expressed in fast muscle (Fig. 4a). EM analysis showed that the diameter of the rescued fast muscle fibers was comparable with that of control muscle (Fig. 4b and Supplementary Fig. S4d). Moreover, swimming velocity, which was significantly decreased in morphants, was partially recovered by the introduction of human mysterin into fast muscle (Supplementary Fig. S4e). Thus, the fast muscle malformation is restored by the introduction of exogenous human mysterin into fast muscle, indicating that human mysterin functionally complements zebrafish mysterin-α and that mysterin-α regulates fast muscle development in a cell-autonomous manner. These observations further ensure that the phenotype that we observed here is not a result of potential off-target effect of morpholino, although the reliability of morpholino technique is controversial in some cases.

We have previously demonstrated that suppression of mysterin-α leads to abnormalities in motoneurons and blood vessels^1^. Are these also cell-autonomous or secondary to fast muscle malformation? We next examined the projection of motoneurons and vascular guidance upon ectopic expression of human mysterin. As noted earlier, mysterin-α morphants exhibited defective projection of primary motoneurons (right panel, Fig. 2b and upper middle panel, Fig. 4c). However, the immature projection of motoneurons was ameliorated by ectopic introduction of human mysterin into fast muscle (white arrows, Fig. 4c and Supplementary Fig. S5), while a few motoneurons failed to form normal projection (upper right panel, Fig. 4c), suggesting that the impaired motor projection is secondary to fast muscle malformation. Notably, mysterin suppression caused a slight developmental delay (Supplementary Fig. S2a,b), which was represented by the increase in synaptotagmin labeling in the spinal cord^7^ (Fig. 4c). Although axonal projection was recovered by fast muscle-specific expression of human mysterin, synaptotagmin labeling in the spinal cord remained intense (Fig. 4c, right panel), suggesting that fast muscle-specific expression of human mysterin does not entirely restore the developmental delay.

We previously demonstrated that the proper patterning of trunk vessels and intracranial vessels is impaired by suppression of mysterin-α using fli:EGFP transgenic zebrafish, which express GFP in vascular endothelial cells^1^. Therefore, we examined whether ectopic expression of human mysterin in fast muscle affects vascular malformation caused by global mysterin suppression in zebrafish larvae (see Methods). As shown previously, vascular guidance was severely impaired by whole-body knockdown of mysterin-α. The formation of blood vessels was not rescued by muscle-specific expression of human mysterin in mysterin-α-suppressed larvae, although fast muscle morphology was restored in the same larvae (Fig. 4d and Supplementary Fig. S6). Thus, vascular malformation is not a secondary effect of the muscle defect caused by reduced expression of mysterin-α in fast muscle.

### Muscle pioneer cells (MPCs) are affected by mysterin knockdown

**Figure 5:**
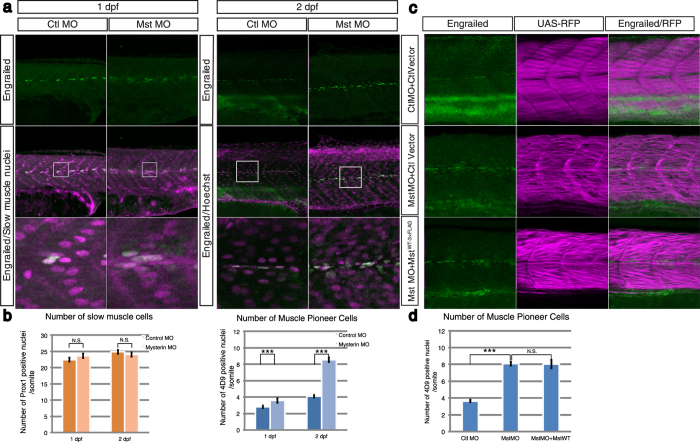
Suppression of mysterin-α significantly increases the number of MPCs. (**a**) Increased number of MPCs upon suppression of mysterin-α. MPCs in the trunk region are stained with an anti-engrailed antibody (green). The nuclei of slow muscle cells at 1 dpf and all cells at 2 dpf are stained with an anti-prox1 antibody (magenta, left panels) and Hoechst (magenta, right panels), respectively. The morphant has a significantly increased number of MPCs at 2 dpf but not at 1 dpf. (**b**) Quantification of slow muscle cells and MPCs. The left panel shows the mean number of nuclei of slow muscle cells (prox1-positive) within 20, 34, 49, and 33 somites of control animals at 1 dpf, mysterin-α morphants at 1 dpf, control animals at 2 dpf, and mysterin-α morphants at 2 dpf, respectively. The right panel shows the mean number of slow muscle nuclei (engrailed-positive) within 43, 52, 20, and 34 somites of control animals at 1 dpf, mysterin-α morphants at 1 dpf, control animals at 2 dpf, and mysterin-α morphants at 2 dpf, respectively. Error bars represent the standard deviation. N.S.: not significant. ***P < 0.001. (**c**) Effect of fast muscle-specific expression of human mysterin on the number of MPCs. Human mysterin tagged with a 3×FLAG epitope (WT-3×FLAG) is expressed in embryonic fast muscle using the Tol2 and GAL4/UAS systems. RFP expression is driven by fast muscle-specific GAL4FF and is thus an indicator of GAL4 activity and fast muscle morphology (See Methods). The number of engrailed-positive nuclei (green) in morphants is unchanged by fast muscle-specific expression of human mysterin. (**d**) Quantification of MPCs shown in (**c**). The panel shows the mean number of nuclei of MPCs (engrailed-positive) within 64, 29, and 25 somites of control animals, mysterin-α morphants, and human mysterin-expressing morphants, respectively, at 2 dpf. Error bars represent the standard deviation. N.S.: not significant. ***P < 0.001.

The slow muscle consists of two subpopulations, authentic slow muscle cells and MPCs^11^,^12^. MPCs are located lateral to the notochord and regulate the orientation of slow and fast muscles and the neuromuscular projections of motoneurons, although their precise mechanisms have not been fully elucidated^13^,^14^,^15^. Therefore, we counted the number of MPCs following immunostaining with an anti-engrailed antibody (4D9), which specifically labels MPC nuclei^16^. The number of engrailed-expressing MPCs in a somite was significantly increased in mysterin morphants (3.6 ± 0.12 at 1 dpf; 8.6 ± 0.29 at 2 dpf) than in control morphants (2.8 ± 0.12 at 1 dpf; 4.1 ± 0.15 at 2 dpf; *P* < 0.001 at both time points) (Fig. 5a,b). On the other hand, the number of slow muscle cells in a somite was unchanged in morphants (Fig. 5b and Supplementary Fig. S7). These data indicate that mysterin-α regulates the differentiation of MPCs. To further elucidate whether fast muscle malformation affects the number of MPCs, we induced expression of exogenous human mysterin in fast muscle of mysterin-α-suppressed embryos. The increase in MPCs was seen in control mysterin knockdown larvae as well as in mysterin morphants that ectopically express mysterin in the fast muscle. These results suggest that the increase in the number of MPCs was not secondary to fast muscle malformation (Fig. 5c,d).

### Functional significance of the AAA+ ATPase and ubiquitin ligase activities of mysterin

**Figure 6:**
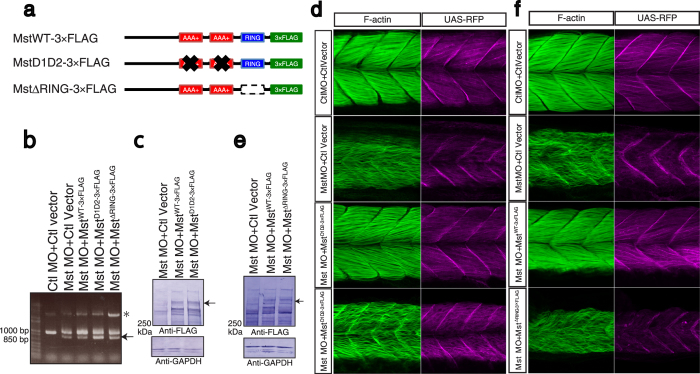
Indispensable roles of the AAA+ ATPase and ubiquitin ligase activities of mysterin. (**a**) Schematic representation of mysterin enzymatic mutants. D1D2 contains four mutations at conserved amino acids in the two AAA+ ATPase modules, resulting in inactivation of ATPase activity and blockade of oligomer formation. ΔRING lacks the entire RING finger domain, which eliminates ubiquitin ligase activity. (**b**) Examination of the knockdown efficiency in morphants by RT-PCR. The lower bands represent the impaired splicing caused by the MO (arrow). (**c**) Ectopic expression of human wild-type and D1D2 mutant mysterin examined by Western blotting using an anti-FLAG antibody. Intact mysterin is indicated by arrow (Also see Supplementary Fig S4b). GAPDH is the loading control. (**d**) Restoration of fast muscle morphology in mysterin morphants by ectopic expression of wild-type human mysterin, but not of the ATPase mutant (D1D2-3×FLAG). Fast actin fibers are labeled with phalloidin (green). RFP expression is driven by fast muscle-specific GAL4FF and is thus an indicator of GAL4 activity. (**e**) Ectopic expression of human wild-type and ΔRING mutant mysterin examined by Western blotting using an anti-FLAG antibody. Mysterin band (591 kDa) is indicated by an arrow (Also see Supplementary Fig S4b). GAPDH is the loading control. (**f**) Restoration of the fast muscle anomaly by expression of wild-type human mysterin, but not of the ubiquitin ligase mutant (ΔRING-3×FLAG). Fast actin fibers are labeled with phalloidin (green). RFP expression is driven by fast muscle-specific GAL4FF and is thus an indicator of GAL4 activity.

The successful reintroduction of human mysterin into zebrafish embryos allowed us to assess the distinct enzymatic functions of mysterin *in vivo*. Although mysterin possesses AAA+ ATPase and RING finger ubiquitin ligase domains and exhibits enzymatic activities^2^, the biological significance of these activities *in vivo* remains to be elucidated. We suppressed expression of endogenous mysterin-α using the splicing-blocking MO and reintroduced wild-type or enzymatically deficient human mysterin into fast muscle (Fig. 6a–c,e). The D1D2 mutant harbors four point mutations that eliminate AAA+ ATPase activity, while the ΔRING mutant has a deletion of the entire RING finger domain, abolishing the ubiquitin ligase activity^1^,^2^. Our rescue assay revealed that neither of these mutants rescued the fast muscle malformation of morphants, which clearly demonstrates that both ATPase and ubiquitin ligase activities are indispensable for the *in vivo* function of mysterin (Fig. 6d,f).

## Discussion

We previously isolated mysterin as the first identified genetic risk factor of human moyamoya disease^1^. A point mutation of mysterin (R4810K) drastically elevates the risk of moyamoya disease; however, the physiological role of mysterin and the functional effect of the R4810K mutation remain largely unknown. In addition to its pathological relevance, mysterin possesses unique and interesting features; it is a huge protein (591 kDa) and the only known protein that possess both AAA+ ATPase and ubiquitin ligase domains. AAA+ ATPases are so-called mechanoenzymes and mediate various physical processes such as membrane fusion and cargo transport through their energy-dependent structural changes. Mysterin forms a toroidal oligomer, a typical structural characteristic of AAA+ ATPases, and dynamically changes its structure through ATP binding and hydrolysis^2^. RING-finger ubiquitin ligases are involved in a broad range of biological processes including proteasomal protein degradation. The pathological relevance of mysterin and how it coordinates these two characteristic activities for its biological functions are intriguing subjects.

In this study, we demonstrated the functional significance of mysterin during early organogenesis of zebrafish. Mysterin-α is indispensable for the proper formation of fast muscle and differentiation of MPCs, while it is likely dispensable for the formation of slow muscle. Suppression of the mysterin-α gene led to the impaired projection of primary motoneurons, although this appears to be a secondary effect of fast muscle malformation. Furthermore, our rescue analyses demonstrated that the two enzymatic activities of mysterin, AAA+ ATPase and ubiquitin ligase activities, are essential for the *in vivo* function of mysterin. This is the first physiologically relevant demonstration that both enzymatic activities are essential for mysterin function.

Mysterin-α-suppressed animals had significantly thinner muscle fibrils in fast muscle due to a significant reduction in the number of myofibrils, while myofibril formation itself appeared to be normal (Fig. 3 and Supplementary Fig. S3a). On the other hand, thinner muscle fibrils were not observed in slow muscle. This suggests that mysterin-α is not involved in muscle fiber formation but rather contributes to fast muscle-specific events such as terminal differentiation and maturation following myogenesis. In addition, mysterin-α-suppressed animals had a significantly increased number of MPCs, a subset of slow muscle cells (Fig. 5), although authentic slow muscle cells were not affected. The fates of fast muscle cells, authentic slow muscle cells, and MPCs are determined before segmentation^11^,^12^. Slow muscle precursors, which are referred to as adaxial cells, are distributed at the most medial area in the segmental plate along either side of the notochord. After segmentation, they execute inside-out migration through myotomal cells toward the superficial area and form a layer of slow muscle cells beneath the skin^17^. A subset of adaxial cells remains motionless and differentiates into MPCs lateral to the notochord. The remaining myotomal cells distributed lateral to the spinal cord and notochord give rise to fast muscle cells. Thus, mysterin-α suppression likely affects fast myogenesis and MPC differentiation after the migration of slow muscle. A part of abnormal morphology of the fast muscle in the morphant was possibly caused by impaired regulation of MPCs.

How does mysterin protein contribute to myogenesis in zebrafish at specific stages? Specific expression of human mysterin in fast muscle cells was sufficient for recovery of the fast muscle morphology. This suggests that mysterin-α plays a physiological role in a cell-autonomous manner. Previous studies elucidated that slow muscle development is regulated through intercellular signaling mediated by multiple humoral factors including sonic hedgehog, bone morphogenic protein, and their receptor molecules^11^,^18^. We previously reported that suppression of mysterin-α leads to excess angiogenesis and abnormal vascular guidance in zebrafish, which partly resemble the phenotypes associated with vascular endothelial growth factor, delta-like 4, plexin D1, and their receptors^1^. Mysterin-α is possibly involved in intercellular communication mediated by humoral factors and receptors. In fact, many AAA+ ATPases and ubiquitin ligases contribute to the cell surface expression and endocytosis of receptors and ligands^4^,^19^. Although its intracellular function remains largely unclear, one possible mechanism is that mysterin-α regulates the secretion or internalization of cell surface molecules and contributes to intercellular signaling. Alternatively, mysterin might be involved in the regulation of transcriptional processes, despite that mysterin is distributed in the cytoplasm.

Is it possible to directly connect the neuromuscular phenotypes with moyamoya disease? Moyamoya disease is characterized by progressive arterial stenosis and hemorrhage from collateral small vessels in limited intracranial sites. These pathological conditions are distinct from the phenotype induced by knockdown of mysterin in zebrafish, in which excess angiogenesis and abnormal vascular patterning are seen. In addition, muscular abnormality has not been reported in human moyamoya disease. Thus, the symptoms of human moyamoya disease and the phenotype of zebrafish with mysterin gene suppression differ. Although previous trials detected no apparent effect of the R4810K SNP on the stability, subcellular distribution, oligomerization, or ubiquitin ligase activity of mysterin^1^,^2^, it is clear that the R4810K SNP exclusively elevates the risk of moyamoya disease by more than 100-fold. Given the dominant inheritance of R4810K-associated moyamoya disease and the difference between moyamoya disease symptoms and the zebrafish phenotype, this SNP possibly induces gain-of-function, rather than loss-of-function, of mysterin.

Another complicating issue is that mysterin-knockout mice exhibit limited phenotypes compared with mysterin morphant zebrafish; the former display slow progression of diabetic symptoms and slight enhancement of post-ischemic angiogenesis^20^,^21^,^22^,^23^. Such phenotypic differences suggest the existence of compensatory pathways in higher vertebrates. Therefore, zebrafish could be a better vertebrate model to investigate mysterin function *in vivo*. Future studies may elucidate the molecular basis on which mysterin contributes to zebrafish organogenesis and the corresponding pathophysiological role of mysterin in higher vertebrates.

## Methods

### Animals

All animal experiments were approved by the institutional animal care and use committee of the National Institute of Genetics and were conducted in accordance with the institutional and national guidelines and regulations. We generated a zebrafish transgenic line *Tg (gSA2AzGFF598A)* by the Tol2 transposon-mediated gene trap approach. In gSA2AzGFF598A, splicing acceptor, the modified Gal4 (Gal4FF) and SV40 polyA signal was integrated in the LIM domain-binding 3b (*ldb3l*) gene, and the Gal4FF was expressed specifically in the fast muscle fibers, mimicking the endogenous *ldb3l* expression pattern. Fast muscle specific expression of Gal4FF was confirmed using the *Tg (UAS:RFP)* transgenic line, which showed RFP expression in the fast muscle (Figs 4a and 6d,f). The *Tg (UAS:RFP)* transgenic line expresses RFP under the control of the UAS promoter. The *Tg (fli:EGFP)* line was used for visualization of blood vessels^24^.

### Morpholinos (MOs) and reverse transcription (RT)-PCR

The sequences of the control and mysterin-α-targeting MOs were as follows: *RNF213-α*–MO1-A: 5′-ACTCGTTGATGTCTGAAGTGATAAA-3′; *RNF213-α*-MO1-D: 5′-AGCTAGGAGAAAGTCCTACCAATTT-3′^1^. For knockdown, 1.7 ng (low dose) or 5.1 ng (high dose) of MO was injected into wild-type zebrafish embryos or 2.1 ng of MO was injected into transgenic zebrafish at the 1–4 cell stage. For ectopic expression of mysterin, Tol2 transposase RNA and a human mysterin-3×FLAG construct or its control empty vector pT2MUAS (2.5 ng) were co-injected together with the MO at the 1–4 cell stage. To determine the knockdown efficiency, total RNA extracted from MO-injected larvae was subjected to RT-PCR using mysterin-α-specific primers^1^.

### Immunohistochemistry

Embryos were treated with pronase (Roche, Basel, Swiss) to artificially remove chorion at 1 day post-fertilization (dpf) and were raised in the presence of 0.2 mM PTU to block pigmentation (Sigma Aldrich, Missouri, USA). Embryos at 1–2 dpf were fixed in 4% paraformaldehyde (PFA) (Nacalai Tesque, Kyoto, Japan), incubated in 1 mg/mL collagenase (Sigma Aldrich) for 10 min (1 dpf) or 20 min (2 dpf), and subsequently incubated in blocking buffer (Blocking One: Nacalai Tesque). For immunolabeling with an anti-FLAG antibody, embryos were fixed in methanol solution (80% methanol and 20% DMSO) at 4 °C overnight. For immunohistochemistry of cross-section samples, embryos were equilibrated in 15–30% sucrose prepared in phosphate-buffered saline (PBS) (137 mM NaCl, 2.68 mM KCl, 8.1 mM Na_2_HPO_4_, and 1.47 mM KH_2_PO_4_, pH 7.4) and embedded in optimal cutting temperature compound (SFJ, Tokyo, Japan) at −80 °C. Sections of a thickness of 10–20 nm were generated using a cryostat (Leica CM3050S, Wetzlar, Germany). The following antibodies were diluted in Blocking One solution and used for immunostaining: anti-slow myosin (F59, mouse monoclonal, 1/50, Developmental Studies Hybridoma Bank (DSHB)), anti-synaptotagmin (znp-1, mouse monoclonal, 1/100, DSHB), anti-prox1 (rabbit polyclonal, 1/500, AngioBio, Del Mar, CA), anti-engrailed (4D9, mouse monoclonal, 1/500, DSHB), anti-FLAG (M2, mouse monoclonal, 1/500, Sigma Aldrich), Alexa 568-conjugated anti-rabbit IgG (1/2000, Life Technologies, California, UAS), and Alexa 488-conjugated anti-mouse IgG (1/2000, Life Technologies). Alexa Fluor 568 phalloidin (1/1000, Life Technologies), Alexa Fluor 488 phalloidin (1/1000, Life Technologies), and Hoechst 33342 (1/1000, Life Technologies) were used to label actin fibers and nuclei. Fluorescence images were captured using a confocal microscope (Zeiss LSM 700, Jena, Germany).

### Western blotting

In total, 10–20 zebrafish embryos at 2 dpf were homogenized in PBS containing 1% NP40, 2 mM EGTA, and a protease inhibitor cocktail (1/100, Nacalai Tesque) and subjected to SDS-PAGE. The following antibodies were diluted in Can Get Signal solution (TOYOBO, Osaka, Japan) and used for Western blotting: anti-FLAG (1/500, Sigma Aldrich) and anti-GAPDH (6C5, mouse monoclonal, 1/1000, Hy Test Ltd., Turku, Finland).

### EM

Embryos at 2 dpf were anaesthetized in 0.02% tricaine, and their heads and tails were removed with a razor. Embryos were subsequently fixed in 2% PFA and 2% glutaraldehyde prepared in 0.1 M phosphor buffer (PB, pH 7.4) at 4 °C overnight. Thereafter, samples were washed with 0.1 M PB and post-fixed in 2% osmium tetroxide prepared in 0.1 M PB at 4 °C for 3 hr. Samples were dehydrated in graded ethanol solutions (50%, 70%, 90%, and 100%). The schedule was as follows: 50% and 70% ethanol for 30 min each at 4 °C, 90% ethanol for 30 min at room temperature, and four times with 100% ethanol for 30 min each at room temperature. Samples were twice infiltrated with propylene oxide (PO) for 30 min each time and incubated in a 70:30 mixture of PO and resin (Quetol-812; Nisshin EM Co., Tokyo, Japan) for 1 hr. Thereafter, the cap of the tube was left open and PO was volatilized overnight. Samples were transferred to fresh 100% resin and polymerized at 60 °C for 48 hr. The polymerized resins were ultra-thin-sectioned at 70 nm with a diamond knife and an ultramicrotome (Ultracut UCT; Leica, Vienna, Austria). The sections were mounted on copper grids, stained with 2% uranyl acetate at room temperature for 15 min, washed with distilled water, and then stained with Lead stain solution (Sigma Aldrich) at room temperature for 3 min. The grids were observed by transmission EM (JEM-1400 Plus; JEOL Ltd., Tokyo, Japan) at an acceleration voltage of 80 kV. Digital images (2048 × 2048 pixels) were acquired with a CCD camera (VELETA; Olympus Soft Imaging Solution GmbH, Münster, Germany).

### Statistical analysis

Data are expressed as means ± standard deviation. Student’s *t*-test was used to analyze differences between two groups. *P* < 0.01 was considered to indicate statistically significant differences.

## Additional Information

**How to cite this article**: Kotani, Y. *et al.* Neuromuscular regulation in zebrafish by a large AAA+ ATPase/ubiquitin ligase, mysterin/RNF213. *Sci. Rep.* **5**, 16161; doi: 10.1038/srep16161 (2015).

## Supplementary Material

Supplementary Information

Supplementary Movie 1

Supplementary Movie 2

Supplementary Movie 3
