# Supplementary material for: Neuromuscular regulation in zebrafish by a large AAA+ ATPase/ubiquitin ligase, mysterin/RNF213
Source: Sci Rep. 2015 Nov 4;5:16161. doi: 10.1038/srep16161 (PMC4632019; doi:10.1038/srep16161)

Neuromuscular regulation in zebrafish by a large AAA+ ATPase/ubiquitin ligase, mysterin/RNF213

Yuri Kotani[1], Daisuke Morito[1,6,7], Satoru Yamazaki[2], Kazutoyo Ogino[3], Koichi Kawakami[4], Seiji Takashima[5], Hiromi Hirata[3,7], Kazuhiro Nagata[1,6,7]

[1]Laboratory of Molecular and Cellular Biology, Faculty of Life Sciences, Kyoto Sangyo University, Kyoto 606-8397, Japan. [2]Department of Cell Biology, National Cerebral and Cardiovascular Center, Suita 565-8565, Japan. [3]Department of Chemistry and Biological Science, School of Science and Engineering, Aoyama Gakuin University, Sagamihara 252-5258 and Center for Frontier Research, National Institute of Genetics, Mishima 411-8540, Japan. [4]Division of Molecular and Developmental Biology, National Institute of Genetics and Department of Genetics, Sokendai (The Graduate University for Advanced Studies), Mishima 411-8540, Japan. [5]Department of Medical Biochemistry, Graduate School of Medicine, Osaka University, Suita 565-0871, Japan. [6]CREST, Japan Science and Technology Agency, Saitama 332-0012, Japan.

[7]Corresponding author:

Daisuke Morito. Laboratory of Molecular and Cellular Biology, Faculty of Life Sciences, Kyoto Sangyo University, Kyoto 606-8397, Japan. Tel: +81-75-705-3090; Fax: +81-75-705-3121; Email: morito@cc.kyoto-su.ac.jp.

Hiromi Hirata. Department of Chemistry and Biological Science, School of Science and Engineering, Aoyama Gakuin University, Sagamihara 252-5258 and Center for Frontier Research, National Institute of Genetics, Mishima 411-8540, Japan. Tel: +81-42-759-6235; Fax: +81-42-759-6235; Email: hihirata@chem.aoyama.ac.jp.

Kazuhiro Nagata. Laboratory of Molecular and Cellular Biology, Faculty of Life Sciences, Kyoto Sangyo University, Kyoto 606-8397, Japan. Tel: +81-75-705-3090; Fax: +81-75-705-3121; Email: nagata@cc.kyoto-su.ac.jp.

Supplementary information

Supplementary Fig. S1. Amino acid sequence alignment among the enzymatic centers of human and zebrafish mysterin.

(a) Comparison of the first AAA+ ATPase regions. Human mysterin and two orthologues of zebrafish mysterin are denoted as hmysterin, zmysterin-α, and zmysterin-β. Walker A and B motifs are nucleotide- and magnesium ion-binding motifs, respectively. They form an enzymatic center of the AAA+ module and are well conserved in the three homologues. (b) Comparison of the second AAA+ ATPase and RING finger domains of human and zebrafish mysterin. The RING finger domains are conserved in human and zebrafish mysterin, except for a cysteine residue (indicated by arrows) that is changed in mysterin-α and -β.

Supplementary Fig. S2. Morphology and motor function of the mysterin morphant.

(a and b) Observation of mysterin morphants at 3 dpf by stereoscopic microscopy. Development of morphants treated with a high dose of the MO targeting mysterin-α is slightly delayed in comparison with that of control embryos. Control embryos and morphants showed head-trunk angles of 166.4° ± 1.2° (n=14) and 137.1° ± 2.6° (n=10), respectively, suggesting significant delay (approximately 20 hr) in embryogenesis. ***$P < 0.001$. Animals carry the fli:EGFP transgene, although vascular endothelial cells were not assessed in this figure. (c) Additional successive images of embryonic swimming at 60 hpf. Tracked embryos are independent of those shown in Fig. 1c.

Supplementary Fig. S3. Electron micrograph of the slow muscle structure in the mysterin morphant.

(a) Quantification of myofibril numbers in 5 μm^2 square of cross-sectionnal electron micrograph. Control and mysterin MOs show 19.0 ± 1.4 (n=12) and 10.6 ± 1.6 (n=11) myofibrils in 5 μm^2 square, respectively. ***$P < 0.001$. Cross-section (b) and vertical section (c) of transgenic animals (gSA2AzGFF598A) that express Gal4FF in fast muscle. Slow muscle is identified by its distribution and the thickness of the Z-disk. Treatment with a low dose of the MO targeting mysterin-α does not affect slow muscle structures.

Supplementary Fig. S4. Fast muscle-specific expression of human mysterin restores the fast muscle malformation and motor deficits of mysterin morphants.

(a) RT-PCR analysis of the knockdown efficiency (2.1 ng of MO) in

morphants. The lower bands represent the impairment of splicing. (b) The expression level of human mysterin in fast muscle was examined by Western blotting using an anti-FLAG antibody. Protein extracts of Mst-3×FLAG-expressing HEK293 cells[1] was loaded for an indication of mysterin products (591kDa; arrow). Mysterin-derived fragments which are not observed in control lanes are indicated by stars. GAPDH is the loading control. (c) Immunostaining of Mst-3×FLAG that is specifically expressed in fast muscle. Control and human mysterin-expressing morphants are stained using an anti-FLAG antibody (white arrows). A single layer and superimposed 3D image are shown separately. RFP is a marker of GAL4 activity. Human mysterin-3×FLAG is distributed in the cytosol, which is consistent with our previous observation in human embryonic kidney 293 cells[1]. (d) EM of vertical sections of human mysterin-expressing morphants. Fast muscle-specific expression of human mysterin-3×FLAG rescues malformation of muscle fibers. Lower panels are magnified images. (e) Swimming motility (mm/s) of human mysterin-expressing morphants.

Manually measured mean speeds of control animals (n = 13), mysterin morphants (n = 10), and rescued animals (n = 15) at 60 hpf. Reduced motility is partially restored by fast muscle-specific expression of human mysterin-3×FLAG. Error bars represent the standard deviation. ***$P < 0.001$.

Supplementary Fig. S5. Projection of motoneurons is restored by fast muscle-specific expression of human mysterin.

Projection of motoneurons is impaired by the MO targeting mysterin-α and is partially restored by fast muscle-specific expression of human mysterin. Motoneurons are labeled using an anti-synaptotagmin antibody. RFP shows Gal4 activity. White arrows show baseline and restored intersegmental projections. The stronger staining of synaptotagmin at the notochord is due to a developmental delay caused by suppression of mysterin-α and is not restored by fast muscle-specific expression of human mysterin.

Supplementary Fig. S6. A morphological defect of blood vessels in mysterin morphants is not restored by fast muscle-specific expression of human mysterin.

(a) Visualization of the vascular structure in the fliEGFP transgenic line. Although three branches of the nasal ciliary artery (white arrows) drain into the inner optic circle (IOC; yellow arrows) in control animals, multiple aberrant vessels drain into the IOC (red arrows) in morphants. This phenotype is not restored by fast muscle-specific expression of human mysterin. (b) Guidance of trunk vessels is severely impaired by mysyerin-α knockdown (red arrows). This anomaly is not restored by fast muscle-specific expression of human mysterin. RFP shows fast muscle-specific Gal4 activity.

Supplementary Fig. S7. Characterization of a previously unidentified subpopulation of MPCs and the failure to recover MPCs by fast

muscle-specific expression of human mysterin.

(a) Co-staining of slow muscle nuclei (prox1: magenta) and MPC nuclei (engrailed: green) in control embryos at 1 and 2 dpf. Although MPCs are known as a subpopulation of slow muscle cells[2], prox1-negative and engrailed-positive cells appear at 2 dpf. (b) Co-staining of MPC nuclei (engrailed: green) and all nuclei (Hoechst: blue) in control embryos at 2 dpf. The proper nuclear distribution of engrailed is confirmed by co-staining of total nuclei (Hoechst: blue). The number of engrailed-positive cells (MPCs) at 2 dpf was counted. (c) The MO targeting mysterin-α does not alter the number of prox1-positive slow muscle cells at 2 dpf. Quantification is shown in Fig. 5b. (d) MPCs are stained with an anti-engrailed antibody (green). The morphant has a significantly increased number of MPCs at 2 dpf, which is not affected by fast muscle-specific expression of human mysterin. RFP shows fast muscle-specific GAL4 activity.

Supplementary Movie 1. Motor function of the control morphant.

Swimming motility of control morphants (5.1 ng of control MO) at 60 hpf. Tactile stimulation elicited an escape response and the larvae moved out of the frame within 150 ms (Fig. 1c).

Supplementary Movie 2. Motor function of the mysterin morphant.

Swimming motility of morphants treated with a low dose (1.7 ng) of the MO targeting mysterin-α at 60 hpf. Tactile stimulation induced an escape response, but much less than in control morphants.

Supplementary Movie 3. Motor function of the mysterin morphant.

Swimming motility of morphants treated with a high dose (5.1 ng) of the MO targeting mysterin-α at 60 hpf. The tactile-induced response is much lower

than in control morphants and morphants treated with a low dose of the MO (Supplementary Movie 1 and 2).

Reference

1. Morito, D. *et al.* Moyamoya disease-associated protein mysterin/RNF213 is a novel AAA+ ATPase, which dynamically changes its oligomeric state. *Sci Rep* **4**, 4442 (2014).
2. Glasgow, E. & Tomarev, S.I. Restricted expression of the homeobox gene prox 1 in developing zebrafish. *Mech Dev* **76**, 175-178 (1998).

Supplementary Fig. S1a

Mysterin 1st AAA+ domain

```
hmysterin      RHKKLERLCLTLGIPQAT-----DPDKTYELTTDNMLKILAIEMRFRCGI
zmysterinα     RWEKIERLSRVLGIQWPL-----DPDETYELTTDNMLKMLAVHMRFRCGI
zmysterinβ     REDKLQKMSFVVGAEKGCEKGKFDPDPTYELTTDNVMKMLAIHMRFRCEI
                    Walker A
hmysterin      PVIIMGETGCGKTRLIKFLSDLRRGGTNADTIKLVKVHGGTTADMIYSRV
zmysterinα     PVIIMGETGCGKTRLIKFLCEMHRSGVATDNMKLVKVHGGTSSEMIYTKV
zmysterinβ     PVIIMGETGCGKTRLVRFLCDLQREGRDVENMKLVKVHGGTTSETIYKKV
                                   Walker B
hmysterin      REAENVAFANKDQHQLDTILFFDEANTTEAISCIKEVLCDHMVDGQPLAE
zmysterinα     REAEAMALRNKLDYGFDTVLFFDEANTTEAISSIKEILCDNSAEGQNLTE
zmysterinβ     REAEELAQKNRQKYKLDTVLFFDEANTTEAIFAIKEVLCDKTVKGYPLKK

hmysterin      DSGLHIIAACNPYRKHSEEMICRLESAGLGYRVSMEETADRLGSIPLRQL
zmysterinα     NTGLKIIAACNPYRKHTDVMIKRLESAGLGYRVRAEETDEKLGSIPLRQL
zmysterinβ     NSGLKIIAACNPYRRHTTKMVDRLERAGLGYRVKAEETEDRLGKVPMRQL

hmysterin      VYRVHALPPSLIPLVWDFGQLSDVAEKLYIQQIVQRLVESISLDENGTRV
zmysterinα     VYRVQALPPSMIPLIWDFGQLNDHTEKMYIKQIVERVAETHSIDSGYITV
zmysterinβ     VYRVHPLPPSMVPLVWDFGQLSDSTELSYIRQIVKKKMRDHRLPLSCQNV

hmysterin      ITEVLCASQGFMRK
zmysterinα     ITDVLSASQKYMRT
zmysterinβ     ITNVLAASQKYMRN
```

Supplementary Fig. S1b

Mysterin 2nd AAA+ domain

```
hmysterin     DSRLLLDEITRAQDLFLDGVPLRKTIAKNLALKENVFMMVVCIELKIPLF
zmysterinα    PMK-VMQEISVIQDLFLGGVPMGENIARNNALKENVFMMVICIELRIPLF
zmysterinβ    SEESLEQEIASCQDFLLKNIQTRETIAKNMALKENVFLMVVCIELRIPLF
                 Walker A
hmysterin     LVGKPGSSKSLAKTIVADAMQGPAAYSDLFRSLKQVHLVSFQCSPHSTPQ
zmysterinα    LVGKPGSSKSLSKTLVADGMQGQAAHSDLFRKLKQIHLVSFQCSPHSTPE
zmysterinβ    LVGKPGSSKSLAKTVIADAMQRQASHCDLFKKLKEVHMVSFQCSPHSSPE
                                        Walker B
hmysterin     GIISTFRQCARFQQGKDLQQYVSVVVLDEVGLAEDSPKMPLKTLHPLLED
zmysterinα    GIINTFKQCARFQEGKNLSEYVSVVVLDEIGLAEDSQKMPLKTLHPLLEE
zmysterinβ    GIIGTFRNCARFQKDKNLDEYVSVVVLDEIGLAEDSPQMPLKTLHPLLED

hmysterin     GCIE-DDPAPHKKVGFVGISNWALDPAKMNRGIFVSRGSPNETELIESAK
zmysterinα    GCID-DQPSPHKKVGFIGISNWALDPAKMNRGIFVSRGDPDENELIESAK
zmysterinβ    GCIDSDNPESYMKVGFVGISNWALDPAKMNRGIFVSRWDPSEKDLVETAE

hmysterin     GICSSDILVQDRVQGYFASFAKAYETVCKRQDKEFFGLRDYYSLIKMVFA
zmysterinα    GICSSDVMILEKVRECFKPFAHAYLRICKKQGKGFFGLRDYYSLIKMMFA
zmysterinβ    GICSSSQPVLLKIKHLLSKLAKCFLSICKTDSEQFFGLRDYYGLIKMLFD

hmysterin     AAKASNRKPSPQDIAQAVLRNFSG-KDDIQALDIFLANLP
zmysterinα    VAKACDQKPSAEQIVKAVLRNFSG-KDDVDAVTFFTSRLN
zmysterinβ    TVKCSDQEPSDKELAEAVLRNFSGQRDGFDPLDYFKDIFQ
```

Mysterin RING finger domain

```
hmysterin     FGIQPCSICLGDAKDPVCLPCDHVHCLRCLRAWFASEQMI-CPYCLTALP
zmysterinα    YGLQLCPVCMGDPRDPLSLPCDHIYCLTCIRQWLVPGQMH-CPLCVQEVP
zmysterinβ    YGVK-CRVCLMELSEPFALPCEHVFCRSCLRRSMEREEAQHCPVCREPLS
```

Supplementary Fig. S2

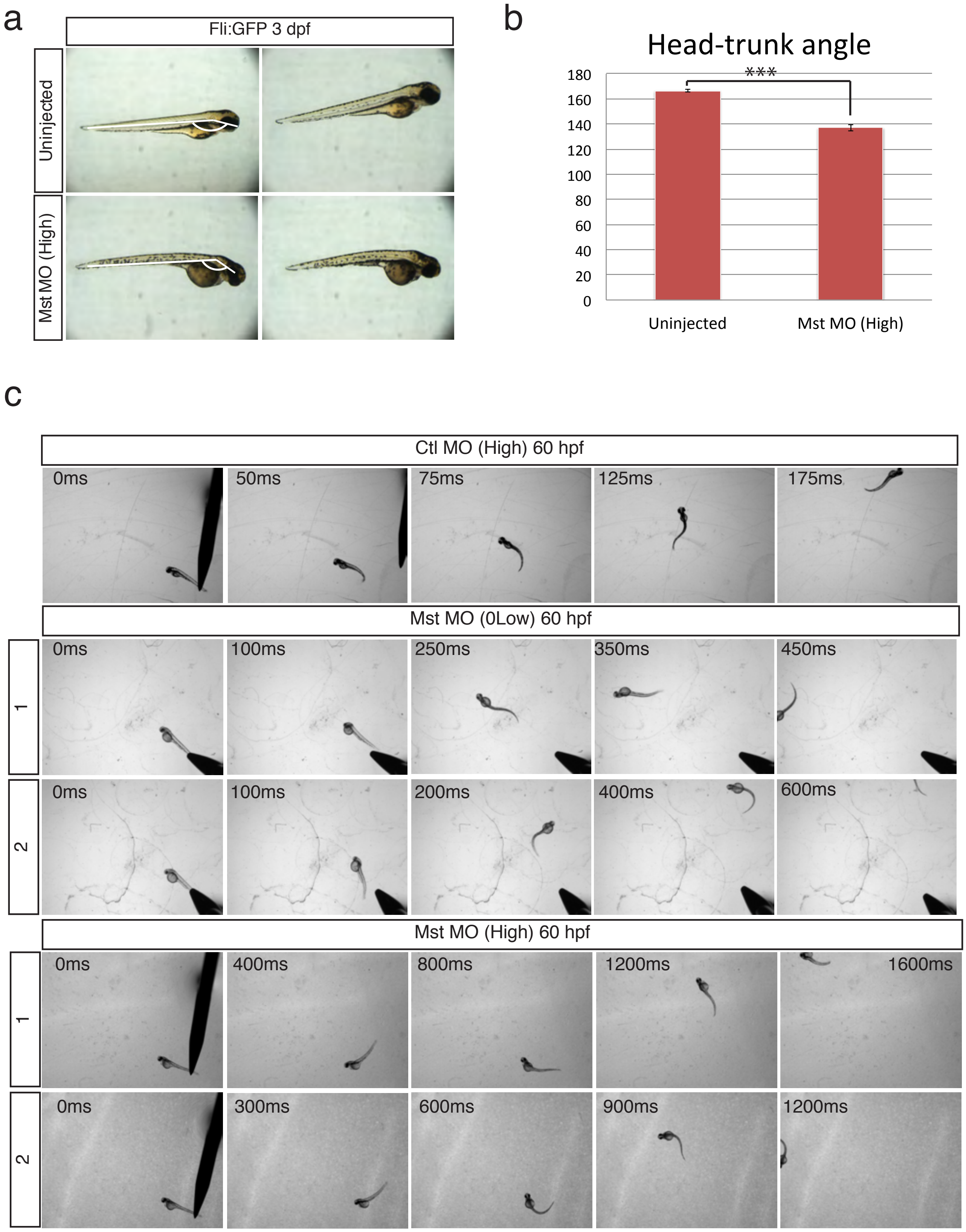

Supplementary Fig. S3

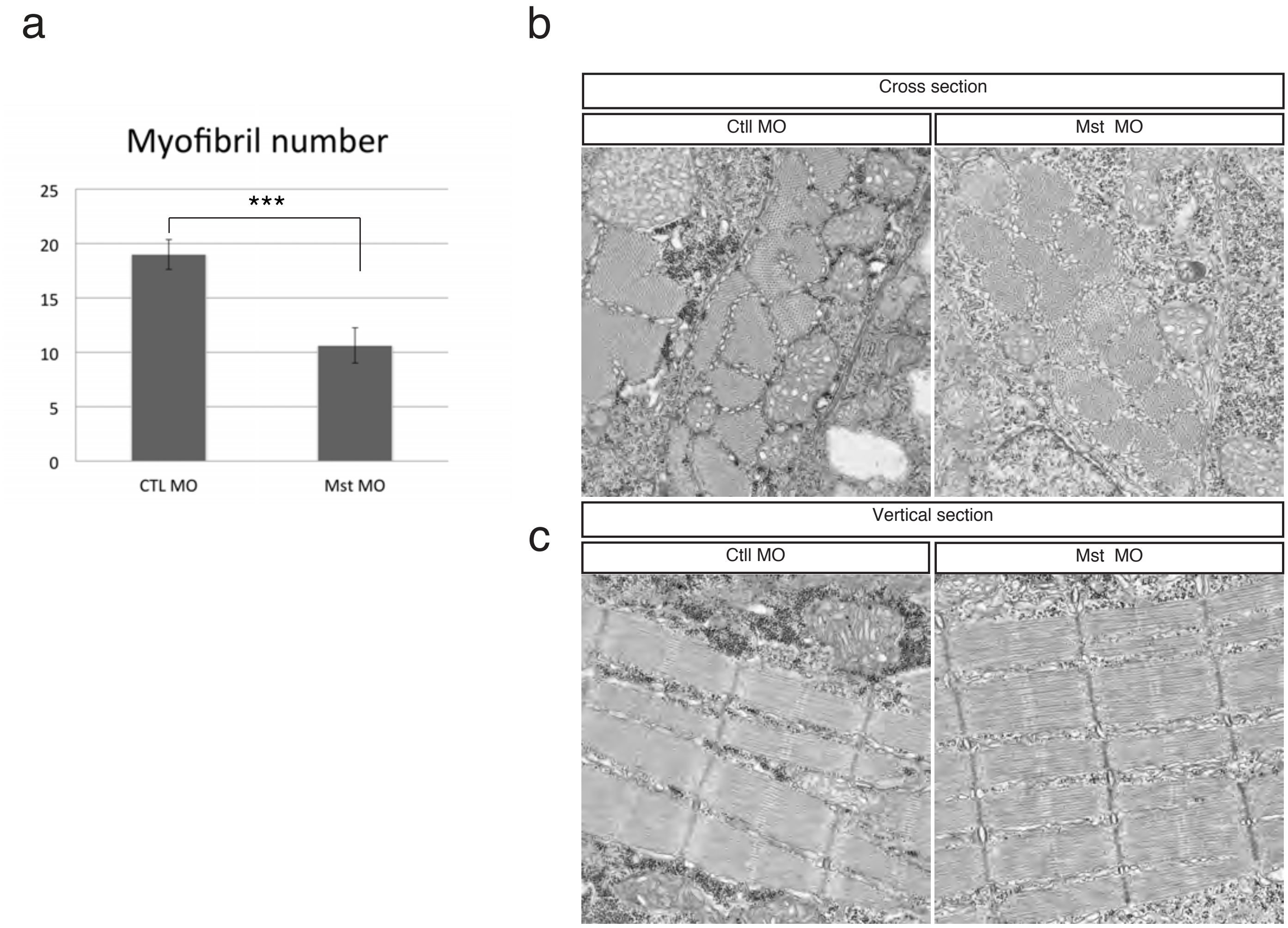

Supplementary Fig. S4

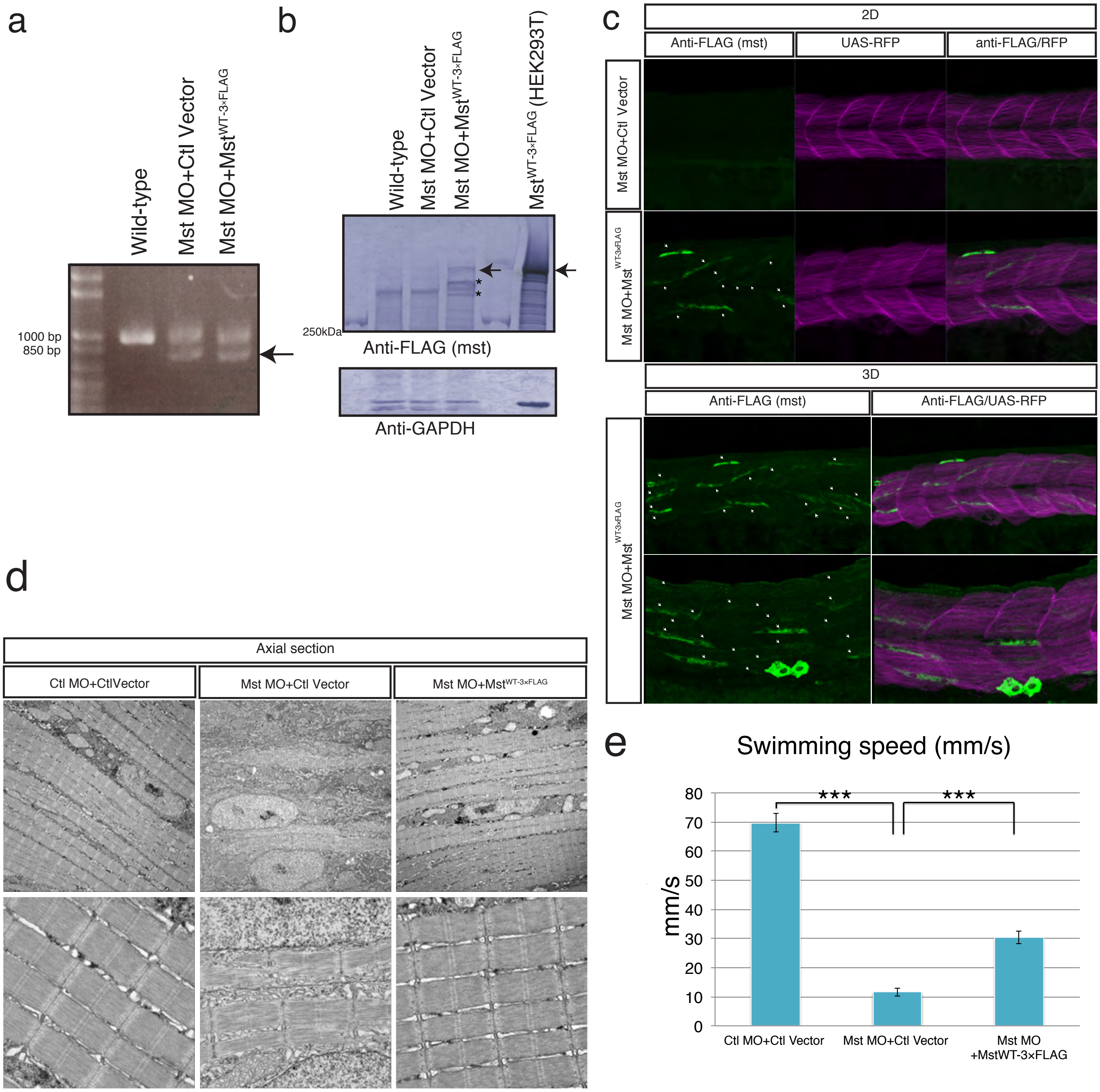

Supplementary Fig. S5

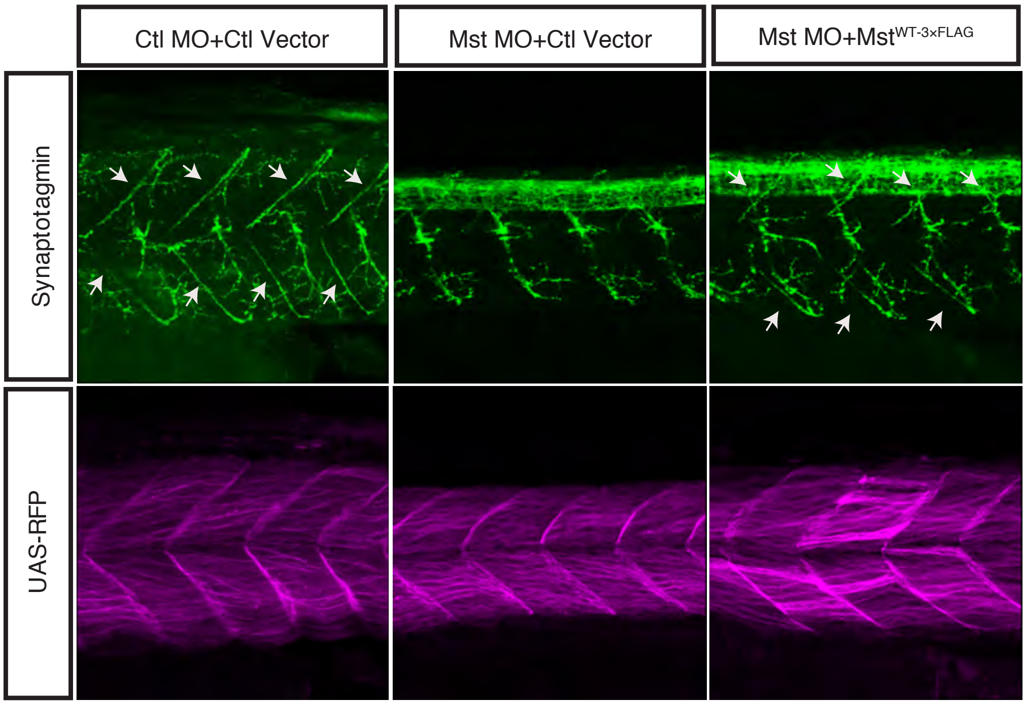

Supplementary Fig. S6

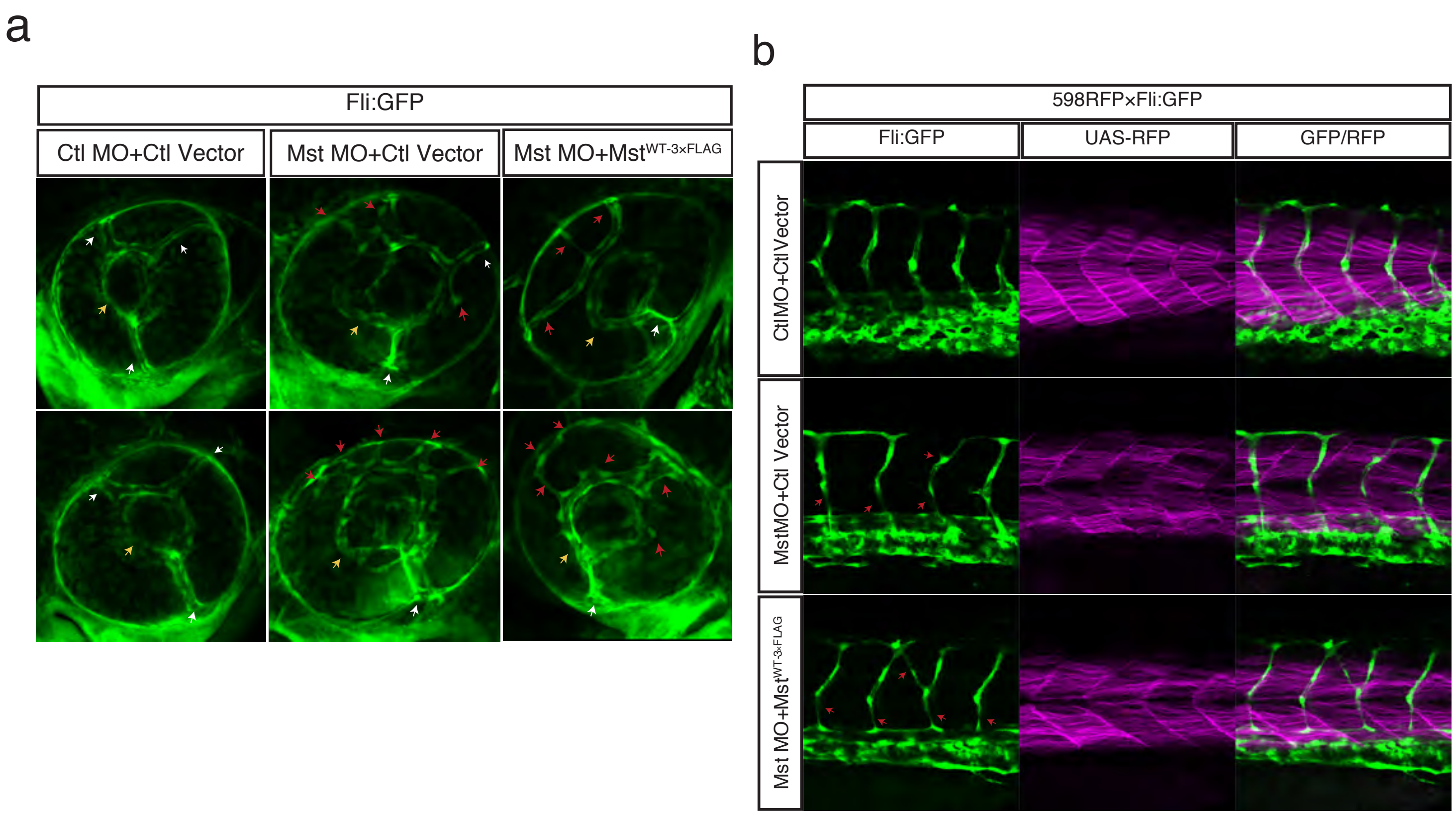

Supplementary Fig. S7

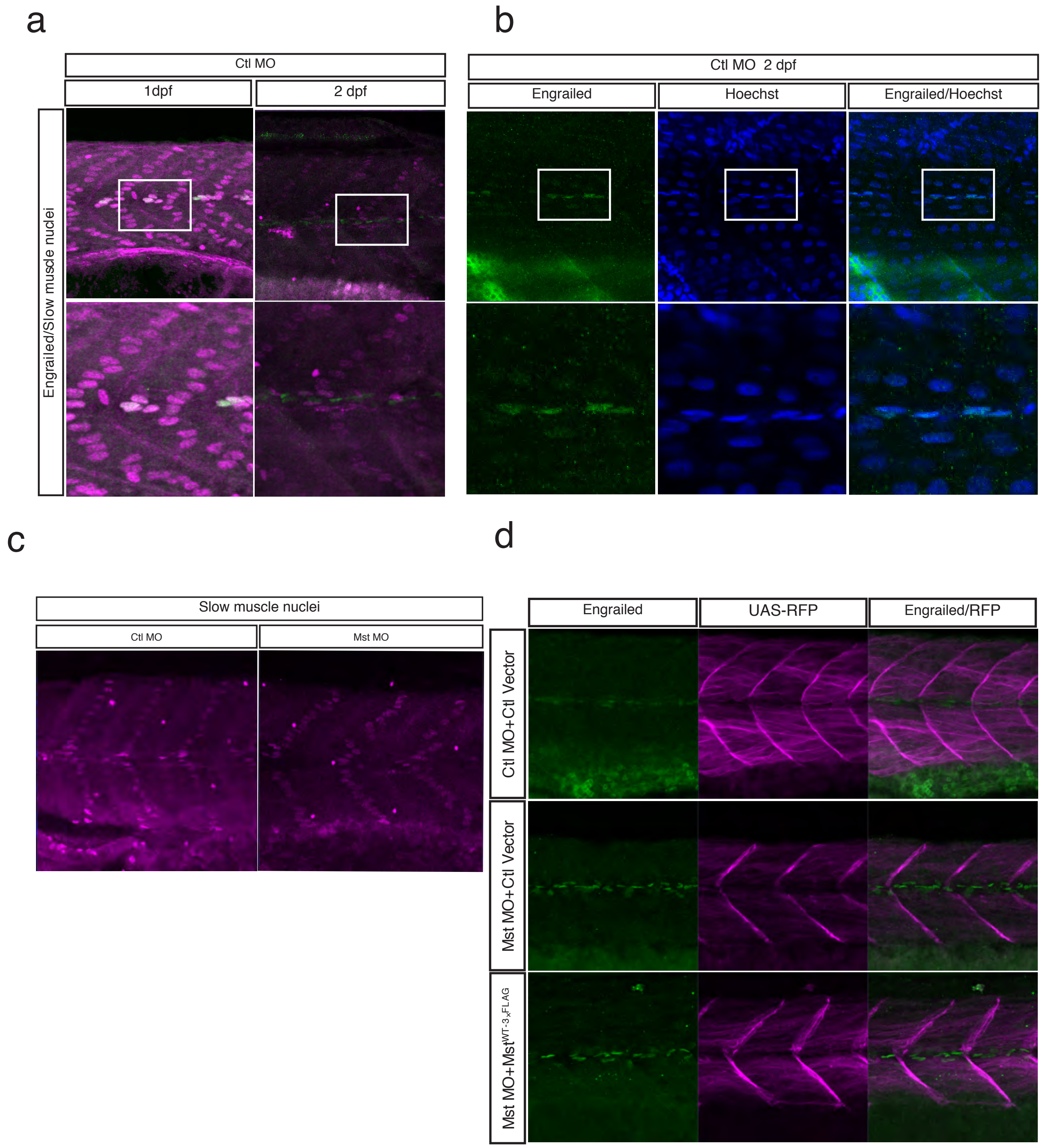

Supplement: Supplementary Information [file srep16161-s1.pdf]
